# Establishment of induced pluripotent stem cell line TRNDi045-A-38 carrying homozygous DOK7-related Congenital Myasthenia patient-mutation knock-in variant from parental KOLF2.1J

**DOI:** 10.1016/j.scr.2026.104090

**Published:** 2026-08-26

**Authors:** B. Malique Jones, Miao Xu, Jizhong Zou, Chengyu Liu, Yaqun Zou, Pankaj Pathak, Richa Madan Lomash, Rodica Stan, Carsten G. Bönnemann, Catherine Z. Chen

**Affiliations:** a Therapeutic Development Branch, National Center for Advancing Translational Sciences, Rockville, MD 20850, USA; b National Heart, Lung, and Blood Institute, USA; c Neuromuscular & Neurogenetic Disorders of Childhood, Neurogenetics Branch, National Institute of Neurological Diseases and Stroke, National Institute of Health, Bethesda, MD 20892, USA

## Abstract

DOK7-related Congenital Myasthenic Syndrome (CMS) is a rare genetic neuromuscular junction disorder. This is one of the most common of the recessive forms of CMS, often presenting with more static proximal weakness (hence also referred to as limb girdle CMS). Whole-genome sequencing of affected patients implicates frameshift duplication mutations in *DOK7* as drivers of impaired neuromuscular-junction signaling. In this study, we generated a human induced pluripotent stem cell (hiPSC) line TRNDi045-A-38 from the KOLF2.1J reference line, engineered to carry homozygous *DOK7* c.1124_1127dupTGCC mutation knock-in using CRISPR/Cas9. This iPSC line could be used for in vitro disease modeling to study disease pathophysiology and for therapeutic development.

## 1. Resource table

**Table:** 

Unique stem cell line identifiers	TRNDi045-A-38

Alternative name(s) of stem cell lines Institution	NCATS-CL4510 National Center for Advancing Translational Sciences, National Institutes of Health, Bethesda, Maryland, USA
Contact information of the reported cell line distributor	Catherine Chen, catherine.chen@nih.gov
Type of cell lines	*iPSC*
Origin	*Human*
Additional origin info *(applicable for human ESC or iPSC)*	*Age: 55–59* *Sex: MaleEthnicity/other genetic background information available: Caucasian; British*
Cell Source	Dermal fibroblasts
Method of reprogramming (if applicable)	Nonintegrating Sendai virus
Evidence of the reprogramming transgene loss (including genomic copy absence/removal/silencing, if applicable) (if applicable)	Pantazis CB et al. Cell Stem Cell.2022; (12):1685–1702. https://pubmed.ncbi.nlm.nih.gov/36459969/
The cell culture system used	Manual/enzymatic-free passaging (EZ Lift Stem Cell Passaging Reagent), maintained in StemFlex™ Medium
Genetic Features: Introduced and Pre-Existing	Homozygous nonsense genetic variant, *DOK7* c.1124_1127dupTGCC (p.Ala378Serfs*30)
Associated disease	*Myasthenic syndrome, congenital, 10; CMS10 OMIM code: 254300*
Gene/locus modified in the reported transgenic lines	*Gene: DOK7* *Locus: 4p16.3* *OMIM number: 610285* *Mutation: homozygous variant DOK7* c.1124_1127dupTGCC (p. Ala378Serfs*30) *(knock-in frameshift duplication in exon 7)*
Method of modification / user-customizable nucleases (UCN) used, *in silico* resources used for design optimization	CRISPR/Cas9
User-customizable nuclease (UCN) delivery method	Nucleofection/electroporation
All double-stranded DNA genetic material molecules are administered to the cells	Cas9 protein, single guide RNA (sgRNA)
Evidence of the absence of random integration of any plasmids or dsDNA introduced into the cells	*N/A*
Analysis of the resulting nuclease-targeted allele status	Fig. 1A
Homozygous allele status validation	Sanger sequencing showed heterozygous C/T SNP downstream of TGCC homozygous knock-in site, confirming homozygous allele status.
Method of the off-target nuclease activity prediction and surveillance	To minimize potential off-target nuclease activity, the CRISPOR program (https://crispor.gi.ucsc.edu/) for guide RNA selection with higher specificity scores and fewer predicted off-targets sites differing from the intended target by at least 1 bp mismatch, and a high-fidelity Cas9 nuclease was employed as a part of the knock-in design approach. Dedicated experimental off-target surveillance was not performed.
Descriptive name of the transgene	N/A
Eukaryotic selective agent resistance cassettes (including inducible, gene/cell type-specific)	N/A
Inducible/constitutive expression system details	N/A
Date archived/stock creation date	*Date cell lines archived or deposited in a repository*
Cell line repository/bank	https://hpscreg.eu/cell-line/TRNDi045-A-38
Ethics/GMO work approvals	Pantazis CB et al. Cell Stem Cell.2022; (12):1685–1702. https://pubmed.ncbi.nlm.nih.gov/36459969/ KOLF2.1J: https://www.jax.org/jax-mice-and-services/ipsc/cells-collection/JIPSC001000
Disclaimers for Addgene/repository-sourced used recombinant DNA resources (if applicable)	N/A

The description of the expected contents of manuscript sections:

## 2. Resource utility

TRNDi045-A-38 is a human induced pluripotent stem cell (hiPSC) carrying a homozygous variant of the downstream of tyrosine kinase 7 (*DOK7)* gene, that is known to be pathogenic and prevalent in DOK7-related congenital myasthenic syndrome (CMS) patients. This line could be differentiated into the myogenic lineage for DOK7-related CMS disease modelling, therapeutic development, and screening small molecule compound libraries.

## 3. Resource details

DOK7-related CMS is a rare genetic disorder of the neuromuscular junction (NMJ) that is clinically observed in patients as “limb-gridle” pattern of proximal muscle weakness (Fernandes et al.,2021, Ohno et al., 2023, Lorenzoni et al.,2020).DOK7 functions primarily to activate muscle-specific tyrosine kinase (MuSK), which allows for the subsequent clustering of acetylcholine receptors in postsynaptic muscle cells. DOK7 deficiency affects a regulator of AChR assembly, and thereby the development and maintenance of the NMJ, leading to altered NMJ signalling (Engel et al., 2015, Liewluck et al., 2011, Oury et al., 2021). A frequently occurring pathogenic mutation in ~15% of DOK7-related CMS patients is the recessive c.1124_1127dupTGCC variant, which causes premature termination and truncation of the C-terminal domain of DOK7, resulting in loss of protein function at the neuromuscular junction (Abicht et al., 2021, Oury et al., 2021, Ziaadini et al., 2024).

To generate an *in vitro* disease model for DOK7-related CMS, we used the well-characterized hiPSC line KOLF2.1J as a standardized cell line for neurological disease (Pantazis et al., 2022), and homozygous inserted a common pathogenic variant causing DOK7-related CMS via CRISPR/Cas9 knock-in. The homozygous c.1124_1127dupTGCC DOK7 knock-in (Fig. 1A) hiPSC line is a valuable resource for disease modeling. This line holds the potential for directed differentiation into myogenic lineages to elucidate cell-autonomous DOK7 functions, as well as for the development of complex skeletal muscle and motor neuron co-culture systems to study NMJ dynamics. The advantage of a knock-in mutation insertion approach is the availability of the parental KOLF2.1J line as an isogenic control. These in vitro models could bypass limitations of *in vivo* studies such as fatality, seen as a severe phenotype involving this variant in rodent models (Webster et al., 2020, Oury et al., 2021, Zhang et al., 2023, Cossins et al., 2025).

The TRNDi045-A-38 line exhibited normal hiPSC colony morphology under phase contrast microscopy (Fig. 1B) and expressed the pluripotency markers SOX2, OCT4, NANOG, SSEA4 and TRA-1–60 by immunofluorescence (Fig. 1C). When examined by flow cytometry, TRNDi045-A-38 cells demonstrated high expression rates for SOX2 (97.6%), OCT4 (98.9%), NANOG (97.3%), SSEA4 (99.7%), and TRA-1–60 (99.2%) (Fig. 1D). G- banding showed a normal karyotype in this line (46, XY) (Fig. 1E), Differentiation potential to the three germ layers was demonstrated in a teratoma formation assay (Fig. 1F). Trilineage differentiation further confirmed the pluripotency of the iPSC line, as the expression of ectodermal (PAX6), endodermal (SOX17), and mesodermal (Brachyury) lineage-specific genes were upregulated in differentiated cells compared with the undifferentiated iPSC line (Fig. 1G). A mycoplasma test confirmed pathogen free status of cells.

## 4. Materials and methods

### 4.1. hiPSC culture and CRISPR engineered knock-in of patient pathogenic variant

hiPSCs were cultured on Geltrex^™^(Gibco^™^ Basement Membrane Matrix, LDEV-free, ThermoFisher Scientific, A1413301) 6-well plates in StemFlex^™^ Medium (ThermoFisher Scientific, A3349401) at 37° C and 5% CO_2_. Cells were passaged at a ratio of 1:6 every 5 days with EZ-LiFT (Millipore Sigma, SCM139). hiPSCs were cryopreserved with CryStor^®^CS10 (Stem Cell Technologies, 07930) and upon initial recovery were cultured with the ROCK inhibitor Y27632 (10 μM; Tocris,1254) overnight.

0.8 million KOLF2.1J iPSCs were transfected with premixed 120 pmol HiFiCas9 V3 (IDT #1081061), 200 pmol sgRNA (Synthego), and 200 pmol ssODN (IDT) using Nucleofector 4D with buffer P3 and program CA-137 (Lonza #V4XP-3024) and then plated onto one well of rhLaminin 521 (Thermo #A29249) coated 6-well plate with StemFlex (Thermo #A3349401), RevitaCell (Thermo #A2644501) and HDR enhancer V2 (IDT #10007921). Transfected cells were cultured in 32°C incubator for 3 days before moving to 37°C incubator, and fresh medium was changed daily after transfection. Single-cell subcloning was done using manual serial dilution to 1 cell/96-well Matrigel coated plate with StemFlex and CloneR2 (Stem Cell Technologies, #100–0691). 10 days later, single-cell clones were picked and confirmed by genomic PCR and Sanger sequencing using the primers listed (Table 2). Due to the heterozygous G/A SNP in KOLF2.1J’s sgRNA target sequence, sgRNA1 and sgRNA2 were used sequentially to generate homozygous c.1124_1127dupTGCC using gRNA 2 (Fig. 1A). Sequence alignment was performed against the wild-type reference sequence. The same ssODN and PCR primers are used in sequential knock-in experiment.

### 4.2. Immunocytochemistry

hiPSCs (passage 24) were passaged at a ratio of 1:6 from one well (6 well plate) into wells on 96 well plate and were cultured for 48 h. Cells underwent fixation with 4% paraformaldehyde for 30 min at room temperature. Following fixation, cells were permeabilized with 0.5% Triton-X-100 for 15 min and then cells were blocked with cell staining buffer (BioLegend, 420201) for 60 min. Primary antibodies were incubated overnight at 4° C in cell staining buffer. Secondary antibodies were incubated the following primary for 60 min at room temperature in cell staining buffer. Hoechst nuclear staining was incubated for 15 min. Proceeding each incubation cells were washed three times with PBS for five-minute intervals. For additional details can be found in Table 2. Images were acquired at 10X magnification using the confocal microscope, Opera Phenix (Revvity, HH1400100) and image analysis software Signals Image Artist ^™^ (Version 1.4.1.91; Revvity, Hopkinton, MA).

### 4.3. Flow cytometry

For flow cytometry analysis, hiPSCs (passage 24) were dissociated into single cells using the gentle cell dissociation reagent (Stem Cell Technologies, 100–0484). Cells underwent fixation with 4% paraformaldehyde for 30 min at room temperature. Following fixation, cells were resuspended in permeabilized with 0.3% saponin for 10 min at room temperature and were stained with intracellular pluripotent marker fluorophore conjugated antibodies (OCT4, NANOG, and SOX2) for 60 min. Cells stained with cell surface pluripotent markers (SSEA4 and TRA-1–60) were resuspended in antibody solution after fixation (SSEA4 and TRA-1–60) for 60 min. Following each incubation, cells were washed with PBS. After staining, the cells were analysed with Novocyte Quanteon Flow Cytometer System and Software (Aligent Technologies, Santa Clara, California).

### 4.4. Karyotyping

Passage-29 hiPSCs were analysed via karyotyping by Stem Genomics Inc. (Durham,NC), using GTG banding of 20 metaphase spreads at 400–500-band resolution.

### 4.5. Teratoma formation assay

At passage 25, ~1 × 10^7^ cells per iPSC line were resuspended in 400*u* L of media supplemented with 10 mM HEPES (pH 7.4) then 200*u* L of cold Matrigel (Corning, 354277). 150*u* L of this suspension was injected subcutaneously into NSG mice (JAX No.005557;https://doi.org/10.1002/cpz1.866) and the resulting tumors were harvested after 6–8 weeks. The tumors were fixed in 10% neutral buffered formalin then embedded in paraffin, sliced, and stained with eosin and hematoxylin.

### 4.6. Trilineage differentiation and RT-qPCR

Passage-14 hiPSCs were differentiated using the STEMdiff ^™^ Trilineage Differentiation Kit following the 12-well plate format procedure. Ectoderm cultures were harvested on Day 7 and mesoderm and endoderm cultures on Day 5. Total RNA was added to the Reliance One-Step Multiplex Supermix (Bio-RAD) and TaqMan ^®^ Gene Expression Assay reaction mix for one-step RT-qPCR. Data was acquired and analyzed using the Quant Studio 7 Pro System (version 1.8.2) and Diomi ^™^ Design and Analysis RUO 3.1.0. Gene expression was normalized to GAPDH.

### 4.7. Short tandem repeat (STR) DNA profile analysis

STR analysis of hiPSC cells (passage 30) was performed by Cell Line Genetics, Inc (Madison, Wisconsin).

## Supplementary Material

MMC1

MMC2

## Figures and Tables

**Fig. 1.**
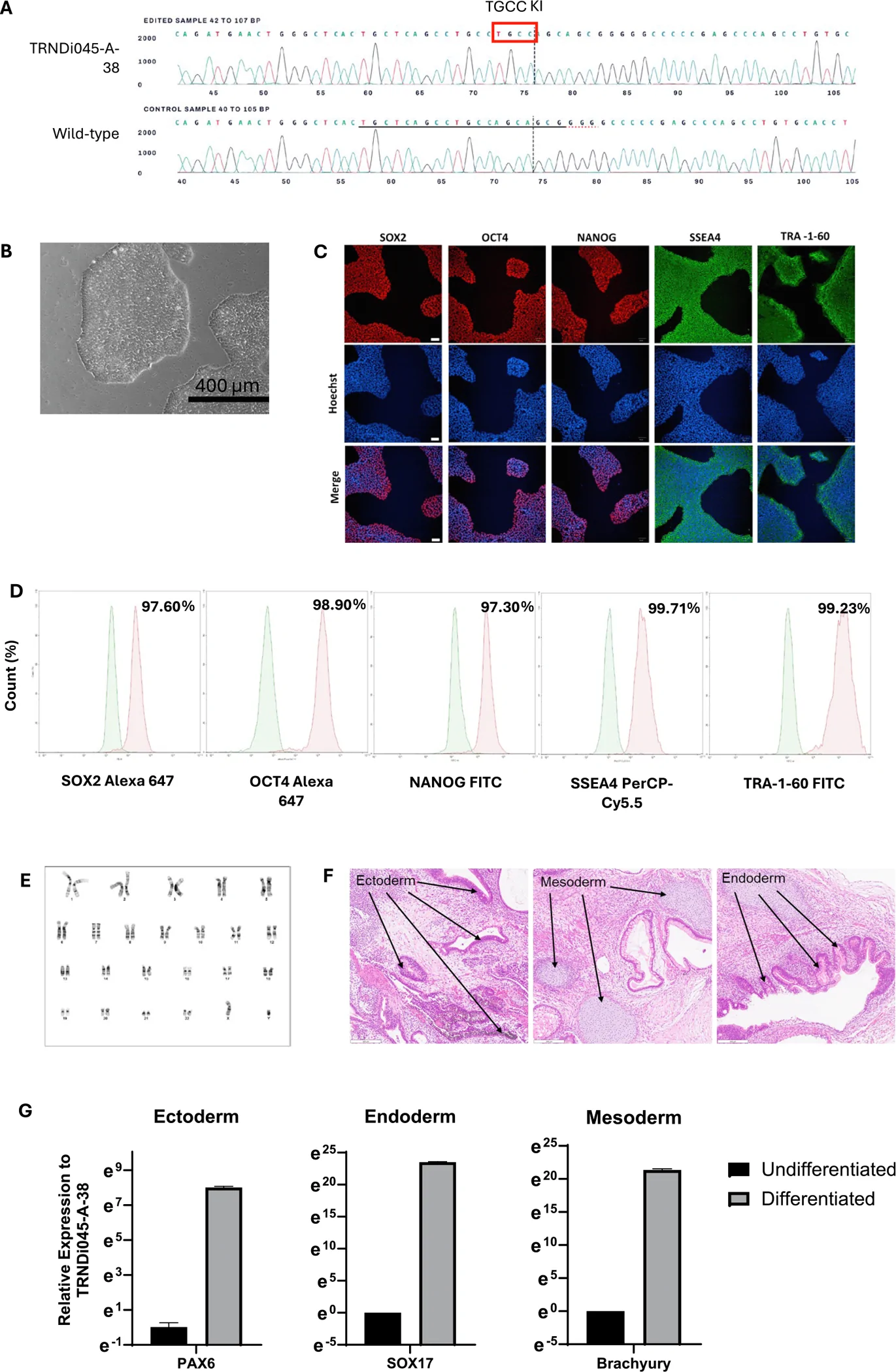
Characterization of TRNDi045-A-38 hiPSC line. (A) Position and detection of editing event for homozygous c.1124_1127dupTGCC variant DOK7 KI. (B) Phase contrast microscopic image at 10X magnification. Scale bar shown in the image. (C) Immunofluorescence images of pluripotency stem cell markers along with nuclear stain (blue). Scale bars shown in the top left figure applies to all panels. (D) Flow cytometry analysis of the positive pluripotency stem cell markers expression. (E) Cytogenetic analysis indicating normal karyotype (46, XY). (F) Immunohistochemistry (teratoma formation assay) showing differentiation of three germ layers (ectoderm, mesoderm, and endoderm). (G) Trilineage differentiation of iPSC line were examined via RT-PCR for ectodermal (PAX6), endodermal (SOX17), and mesodermal (Brachyury) lineage-specific gene expression, compared with the undifferentiated iPSC line.

**Table 1:** Characterisation and validation.

Classification	Output type	Result	Data (suggested placement)

Schematic of a transgene/genetic modification	Schematic illustrating the structure and location of the introduced genetic modification	A visual representation of the edited allele, showing heterozygous SNP knock-in site compared to wildtype.	Fig. 1 panel A
Morphology	Photography Bright Field	Normal	Fig. 1 panel B
Characterization of the pluripotent/ undifferentiated state	Immunocytochemistry	SOX2, OCT4, NANOG, SSEA4, and TRA-1-60	Fig. 1 panels C-D
	Flow cytometry	97.60%(SOX2),98.90% (OCT4),97.30%(NANOG),99.71% (SSEA4), 99.23% TRA-1-60	
Karyotypic characterization	Karyotyping(G-banding)	46XY Banding Resolution: 400-500	Fig. 1 panel E
Genotyping for the desired genomic alteration/allelic status of the gene of interest	PCR across the edited site or/and targeted allele-specific PCR Evaluation of the — (homo-/hetero-/hemi-) zygous status of introduced genomic alteration(s) Transgene-specific PCR (when applicable)	Sanger chromatogram comparing TRNDi043-A-38 sequence to wild-type reference sequence. *N/A* *N/A*	Fig. 1 panel A *N/A* *N/A*
Verification of the absence of random plasmid integration events	PCR/Southern	*N/A*	N/A
Evidence of genetic identity for the parental and the reported genetically-modified cell lines	STR analysis	*DNA Profiling* Short Tandem Repeat (STR) analysis of 20 allele loci showed identical results as parental cell line (Amelogenin + D1S1656, D10S1248, D5S818, D13S317, D7S820, D16S539, TPOX, CSF1PO, Penta E, D16S539, D18S51, D2S1338, Penta D, THO1, D21S11, D7S820, D8S1179,D12S91, D19S433, D22S1045)	*N/A* Supplementary Table 1 Parental cell line (Binder et al., 2024)
Mutagenesis / genetic modification outcome analysis	Sequencing (genomic DNA PCR or RT-PCR product)	Confirmation of the precise nature of the introduced alterations in all described cell lines, also homozygous state of mutation	Fig. 1 panel A
	PCR-based analyses	The homozygous allele status was confirmed by Sanger sequencing showing homozygous SNP downstream knowck- in site (TGCC KI is around nucleotide position 80)	Supplementary Fig. 1
	Southern Blot or WGS; western blotting (for knock-outs, KOs)	*N/A*	*N/A*
Off-target nuclease activity analysis	PCR across top 5/10 predicted top likely off- target sites, whole genome/exome sequencing	N/A	N/A
Specific pathogen-free status	Mycoplasma	Mycoplasma testing by luminescence. Negative	Supplementary Table 2
Characterization of the differentiation potential	Teratoma formation and trilineage differentiation	Teratoma with three germ layers formed: Ectoderm; Mesoderm; Endoderm	Fig. 1 Panel F
		Trilineage differentiation: expression of ectodermal, mesodermal, and endodermal markers	Fig. 1 Panel G
*List of recommended germ layer markers*	Realtime-PCR	Ectoderm: PAX6 Endoderm: SOX17 Mesoderm: Brachyury	Fig. 1 Panel G
*Overall outcomes of gene editing experiment (OPTIONAL)*	Brief description of the outcomes in terms of clones generated / establishment approach / editing event screening outcomes	N/A	N/A
*Donor screening (OPTIONAL)*	HIV 1 + 2 Hepatitis B, Hepatitis C	Refer to Jackson Laboratory	N/A
*Genotype — additional histocompatibility info (OPTIONAL)*	Blood group genotyping HLA tissue typing	Refer to Jackson Laboratory Refer to Jackson Laboratory	N/A N/A

**Table 2:** Reagents details RRID Requirement for antibodies: use http://antibodyregistry.org/ to retrieve RRID for antibodies and include ID in the table as shown in examples.

Antibodies and stains used for immunocytochemistry/flow-cytometry				
		Antibody	Dilution	Company Cat # and RRID

Pluripotency Markers		Mouse anti-SOX2	1:400	Cell Signaling Technology Cat# 4900, RRID:AB_10,560,516
Pluripotency Markers		Rabbit anti- OCT4	1:400	Cell Signaling Technology Cat# 2890, RRID:AB_2,167,725
Pluripotency Markers		Rabbit anti-NANOG	1:200	Cell Signaling Technology Cat# 4903, RRID:AB_10559205
Pluripotency Markers		Mouse anti-SSEA4	1:500	Cell Signaling Technology Cat# 4755, RRID:AB_1264259
Pluripotency Markers		Mouse anti-TRA-1-60	1:500	Cell Signaling Technology Cat# 4746, RRID:AB_2119059
Flow Cytometry		Mouse anti-SOX2	1:50	BD Biosciences Cat# 562139, RRID:AB_10897844
Flow Cytometry		Mouse anti-OCT3/4	1:50	BD Biosciences Cat# 562252, RRID:AB_11153862
Flow Cytometry		Rabbit anti-NANOG	1:50	Millipore Cat# FCABS352A4, RRID:AB_10807973
Flow Cytometry		Mouse anti-SSEA4	1:50	BD Biosciences Cat# 561565, RRID:AB_10894210
Flow Cytometry		Mouse anti-TRA-1-60	1:50	ThermoFisher Scientific Cat#MA1023D488X,RRID:AB_2536700
Secondary antibodies		Donkey Anti-Mouse IgG (Alexa Fluor 488)	1:400	ThermoFisher Scientific Cat# A21202, RRID:AB_141607
		Donkey Anti-Rabbit IgG (Alexa Fluor 594)	1:400	ThermoFisher Scientific Cat# A21207, RRID:AB_141637
Nuclear stain		Hoechst33342	1 μg/mL	Thermo Fisher Scientific Cat# H3570, RRID:AB_3675235
Site-specific nuclease Nuclease information		*Nuclease type/version*	Alt-R ^™^ S.p. HiFi Cas9 Nuclease V3 (IDT #1081061)	
Delivery method		*Electroporation (parameters), lipofection*	Nucleofector 4D with buffer P3 and program CA-137 (Lonza #V4XP-3024)	
Selection/enrichment strategy	*Selection cassette(s), FACS*	No selection/enrichment		
Primers and Oligonucleotides used in this study				

	Target	Forward/Reverse primer (5′-3′)		

Pluripotency Markers (qPCR)	*PAX6*	*Hs00240871_m1*		
	*T:TBXT(T/Brachyury)*	*Hs0061080_m1*		
	*SOX17*	*Hs00751752_s1*		
House-Keeping Genes (qPCR)		*GAPDH*	*ThermoFisher Scientific Cat# 4310884E*	
gRNA oligonucleotide/crRNA sequence		Provide gRNA seed genomic sequence, or full gRNA/crRNA	sgRNA1: TGCTCAGCCTGCCAGCAGCG sgRNA2: TGCTCAGCCTGCCAGCAGCA	
Bioinformatic gRNA on- and –off-target binding prediction tool used, specific sequence/outputs link(s)		CRISPOR	https://crispor.gi.ucsc.edu/	
Primers for top off-target mutagenesis predicted site sequencing (for all CRISPR/Cas9, ZFN and TALENs)		OT1 - F&R	CAGCCACTCCTCTTACTCCA ACATGCCGAAAAGAAAGCCG	
		OT2 - F&R	CAGCCACTCCTCTTACTCCA	
		...	ACATGCCGAAAAGAAAGCCG	
ODNs/plasmids/RNA molecules used as templates for HDR-mediated site-directed mutagenesis.		Backbone modifications in utilized ODNS have to be noted using standard nomenclature.	*ssODN 1:* *ACGTGTGGCGGGCCACAGATGAACTGGGCTCACTGCTCAGCCTGCCTGCCAGCAGCGGGGGCCCCCGAGCCCAGCCTGTGCACCTGCCTGCCCGGGACAG* *ssODN2:* *ACGTGTGGCGGGCCACAGATGAACTGGGCTCACTGCTCAGCCTGCCTGCCAGCAGCGGGGGCCCCCGAGCCCAGCCTGTGCACCTGCCTGCCCGGGACAG*	

## Data Availability

Data will be made available on request.

## Appendix A. Supplementary data

Supplementary data to this article can be found online at https://doi.org/10.1016/j.scr.2026.104090.
