# Supplementary material for: Establishment of induced pluripotent stem cell line TRNDi045-A-38 carrying homozygous DOK7-related Congenital Myasthenia patient-mutation knock-in variant from parental KOLF2.1J
Source: Stem Cell Res. Author manuscript; Available in PMC 2026 Oct 7. (PMC13643754; doi:10.1016/j.scr.2026.104090)
Supplement: Supplementary file 1 [file NIHMS2208064-supplement-MMC2.pdf]

Supplemental Table 1.

TRNDi045-A-38 p20	POSTIVE CONTROL	NEGATIVE CONTROL
0.34 ± 0.019	109.1 ± 13.5	0.14 ± 0.026

>1.2	Mycoplasma Positive
0.9-1.2	Results Ambiguous
<0.9	Mycoplasma Negative
